# Salt marsh vegetation promotes efficient tidal channel networks

**DOI:** 10.1038/ncomms12287

**Published:** 2016-07-19

**Authors:** William S. Kearney, Sergio Fagherazzi

**Affiliations:** 1 Department of Earth and Environment, Boston University, 685 Commonwealth Avenue Boston, Massachusetts 02215, USA

## Abstract

Tidal channel networks mediate the exchange of water, nutrients and sediment between an estuary and marshes. Biology feeds back into channel morphodynamics through the influence of vegetation on both flow and the cohesive strength of channel banks. Determining how vegetation affects channel networks is essential in understanding the biological functioning of intertidal ecosystems and their ecosystem services. However, the processes that control the formation of an efficient tidal channel network remain unclear. Here we compare the channel networks of vegetated salt marshes in Massachusetts and the Venice Lagoon to unvegetated systems in the arid environments of the Gulf of California and Yemen. We find that the unvegetated systems are dissected by less efficient channel networks than the vegetated salt marshes. These differences in network geometry reflect differences in the branching and meandering of the channels in the network, characteristics that are related to the density of vegetation on the marsh.

## Abstract (web summary)

Tidal channel networks mediate the exchange of water, nutrients, sediment and biota between an estuary and marshes. Here, the authors show that the presence of vegetation on the marsh platform contributes to the formation of an efficient channel network.

## Graphical abstract

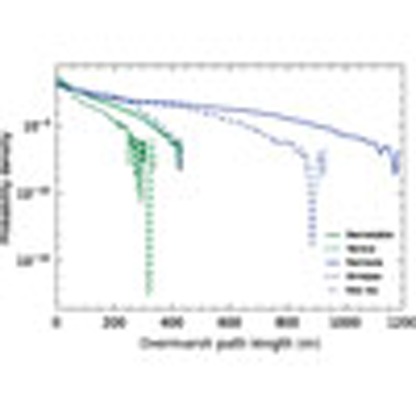

The geometry of tidal channel networks in salt marshes controls the flow of water, nutrients, sediment and biota through intertidal systems which in turn influences the provision of ecosystem services in these systems^1^ such as carbon sequestration^2^ and flood protection^3^,^4^,^5^,^6^. Vegetation has been observed to influence the branching and meandering characteristics of tidal channels^7^,^8^ and has been shown experimentally and numerically to stabilize creek banks, promoting meander formation in fluvial systems^9^,^10^,^11^. Experimental tidal channel networks without vegetation can give rise to meandering channels in certain settings^12^,^13^,^14^, but conclusive experimental proof of the influence of vegetation on tidal channel networks is elusive because of difficulties in scaling the impacts of vegetation on sediment transport to laboratory scales. While models of tidal channel network formation can form meandering channels^15^,^16^, few have yet examined the impact of vegetation on the system-scale geometry of tidal channel networks^17^,^18^. Models focused on channel initiation and development^19^,^20^,^21^ have shown that adding the effects of vegetation on hydrodynamics can lead to enhanced channel formation on tidal flats with vegetation stabilizing the channels as the flats develop into salt marshes, but these models do not usually focus on the late stage of channel development characterized by meander formation and evolution^21^,^22^,^23^.

Marani *et al*.^24^ found that traditional measures of network geometry, such as drainage density^25^, failed to distinguish marsh channel networks with different patterns of meandering and branching. Instead, they propose to use the unchanneled path length^26^, a measure of the distance a drop of water, placed on the marsh surface, would have to travel on the platform before reaching a channel. They also provide a new measure we call the geometric efficiency, which is the Hortonian length (the inverse of drainage density) divided by the mean unchanneled path length for a subbasin of a network. This measure gives an indication of how well a channel network serves the marsh platform. For a given Hortonian length, that is, for a given channel length and basin area, a more efficient channel network means that a larger portion of the marsh platform is close to a channel.

To understand the influence of vegetation on tidal channel network geometry, we compare the channel network geometry of typical salt marshes (Barnstable, Massachusetts, United States and Venice, Italy) with arid, unvegetated systems in Baja California, Mexico and Al Hudaydah, Yemen. We find that the vegetated salt marshes have more efficient tidal channel networks than the unvegetated systems. This difference in the observed network geometry is due to the difference in branching and meandering characteristics of the channels. Branching and meandering characteristics are controlled largely by the cohesiveness of sediment within the system, which is in turn augmented by the presence of vegetation in the salt marshes.

## Results

### Study sites

The arid counterpart of the salt marsh is known as a sabkha^27^. High rates of evaporation concentrate salinity on the surface of sabkhas and lead to the deposition of evaporites^28^. The term ‘sabkha' refers, however, to a gradient of hypersaline environments from subtidal to supratidal flats and inland salt lakes (playas), which vary in their hydroperiod and, consequently, their salinity, biogeochemistry and vegetation status^28^,^29^.

**Figure 1:**
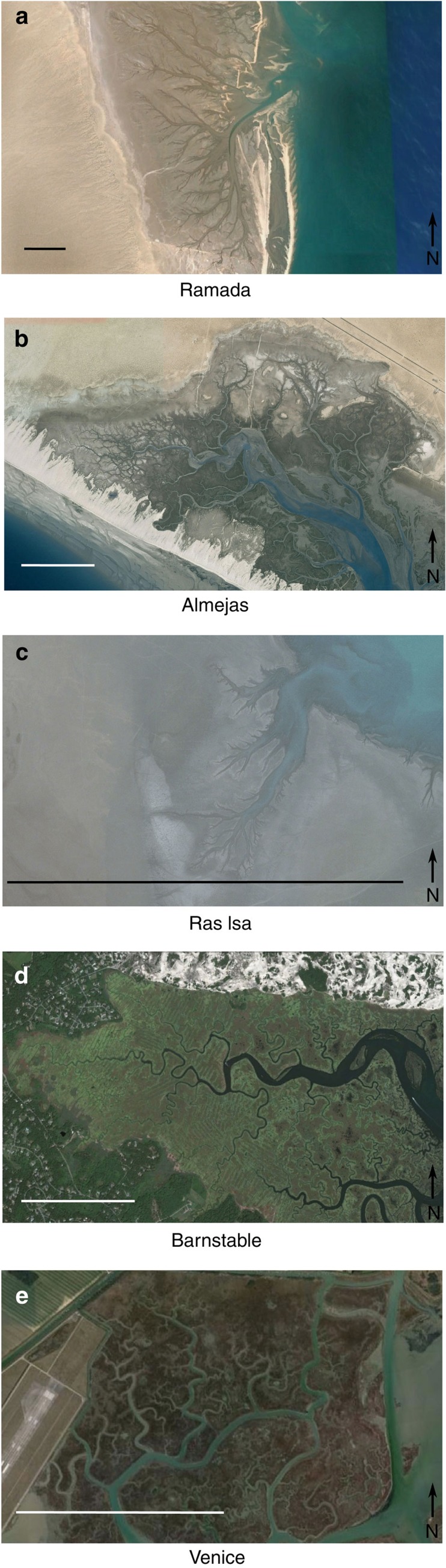
Imagery of channel networks. Satellite imagery of the five intertidal systems studied. The scale bars each represent a length of 1 km. (**a**) Ramada, Baja California, Mexico (Google, Digital Globe; 31.25° N, 114.90° W), (**b**) Almejas, Sonora, Mexico (Google, INEGI, CNES/Spot Image; 31.21° N, 113.13° W), (**c**) Ras Isa, Yemen (Google, CNES/Astrium; 15.24° N, 42.76° W), (**d**) Barnstable, MA, USA (Google; 41.73° N, 70.37° W), and (**e**) Palude Pagliaga, Venice, Italy (Google; 45.51° N, 12.38° W).

We analyse the channel network geometry of a sabkha on the Ras Isa peninsula in the Al Hudaydah governorate, Yemen, and similar hypersaline lagoons called ‘esteros' in the Gulf of California, Mexico^30^. One of the largest of these is the Estero la Ramada, also known as La Bolsa, in Baja California (Fig. 1a). The tidal range in the Gulf of California can reach 9 m, but is closer to 6–7 m near the Estero la Ramada^31^, and the sediments in the Estero la Ramada are fine (clayey silts)^31^. The Normalized Difference Vegetation Index (NDVI) calculated from Landsat 8 imagery shows that Ramada is largely unvegetated in contrast to other intertidal marshes (the mean NDVI of the Ramada platform is 0.05 compared with that of Barnstable, 0.37). Imagery of Ramada shows a network geometry characterized by a dendritic network rather than the meandering channels often seen in vegetated marshes. Another estero, Almejas, on the opposite, Sonoran side of the Gulf of California, shows both classes of network geometry. Vegetated, meandering channels lie relatively close to the inlet and unvegetated, dendritic ones are further up the estero (Fig. 1b). The dendritic channel network geometry characteristic of Ramada is not limited to Gulf of California esteros or to macrotidal coasts as the unvegetated sabkha in Ras Isa, (Fig. 1c) with a tidal range around 1 m, has a channel network, which resembles that of Ramada. Sabkhas on the Arabian coast of the Red Sea tend to have clastic sediments with sandy mud textures^32^,^33^. The sedimentary properties of sabkhas in general depend strongly on the source of the sediment that builds them. They can have widely varying sediment types from sand to fluvial or aeolian silt and clay as well as the characteristic evaporites^28^,^34^,^35^,^36^. Sabkha soils tend to be weak^37^, especially when tidally flooded because of the repeated dissolution and recrystallization of salts within the soil.

Barnstable marsh in Massachusetts, United States, (Fig. 1d) and the Palude Pagliaga in the Venice Lagoon, Italy, (Fig. 1e) represent, respectively, macrotidal and microtidal vegetated salt marshes with a meandering network geometry. The vegetation of Barnstable is dominated by grasses of the genuses *Spartina* and *Distichlis*. *S. alterniflora* occupying lower elevations and more poorly drained sites while *S. patens* and *D. spicata* occupy well-drained higher elevations^38^. Vegetation in the Pagliaga marsh consists of the reed *Phragmites australis*, the rush *Juncus maritimus*, the succulent *Salicornia veneta* and the shrub *Halimione portulacoides*^39^. The sediments of both the Barnstable and Venice lagoon are fine-grained. Elevation data derived from the Shuttle Radar Topography Misson indicate that all five of these systems are flat and lie above mean sea level, but below mean high tide, typical conditions of salt marshes. The five analysed systems, their approximate tidal ranges and their vegetation status are shown for reference in Table 1.

### Network statistics

The values of the relevant network parameters for each of the five systems considered are given in Table 1. The drainage density (*D*) of Barnstable marsh (0.01 m^−1^) is nearly twice that of Ramada (0.007 m^−1^). This corresponds to a Hortonian length (*l*_h_) of 86.63 m for Barnstable and 145.30 m for Ramada. The mean unchanneled path length (*l*) of Barnstable (45.44 m) is a third of that of Ramada (132.98 m). Barnstable has a smaller Hortonian length but a much smaller mean unchanneled path length than Ramada, which leads to a greater geometric efficiency (*l*_h_/*l*) for Barnstable (1.91) than for Ramada (1.09). This difference in efficiency captures the meandering geometry of the Barnstable network and the straight, branching geometry of Ramada. We note the utility of the dimensionless efficiency measure in the case of the Ras Isa system. Because Ras Isa is a smaller network than the others (on the order of one square kilometre), both the dimensional Hortonian length and unchanneled path length are much smaller, closer in value to the vegetated systems. However, the ratio of the two, the efficiency (0.75), shows that Ras Isa is even less efficient than Ramada. The vegetated Venice marsh has an efficiency greater than that of Barnstable (3.15), while the partially vegetated Almejas has an efficiency between that of Ramada and Barnstable (1.41). We tested the differences in efficiency with Mann–Whitney *U*-tests with a Bonferroni correction for multiple comparisons^40^ and found that the differences in efficiency between each system are significant (*P*<0.002) except for the difference between the two vegetated systems Barnstable and Venice.

## Discussion

The geometric efficiency captures the branching and meandering characteristics of the channel network. We find that marshes with highly sinuous channels have relatively high efficiencies because they have relatively small unchanneled path lengths. In these systems like Barnstable and Venice, channels take wide meanders across the surface and so reduce the distance water travels over the marsh surface before reaching a channel. In the low-efficiency systems of Ramada, Ras Isa and the unvegetated portions of Almejas, the channels do not meander, and the unchanneled path length correspondingly increases. The cohesive nature of sediments can generate meanders in streams^41^, but sediment grain sizes are relatively similar between the esteros and the salt marshes systems (though the sediment is sandier in the Ras Isa sabkha), so this phenomenon does not account for these observed differences in network geometry. We invoke the additional bank cohesion provided by vegetation to explain the presence of meandering channels in the Barnstable and Venice marshes and their absence in the Ramada and Ras Isa systems. Such a dependence of the measures of drainage density on vegetation properties has been observed in the evolution of a salt marsh from an unvegetated tidal flat^8^, and we find the same patterns—unvegetated systems have high unchanneled path lengths—in the arid systems examined here.

The geometric efficiency is also a measure of the ability of the channel network to drain the marsh platform. This interpretation of network efficiency may have implications for the biogeochemical cycling in a marsh and for its ecological functioning. Soil water drainage is responsible for the observed increase in productivity of halophytic vegetation (for example, *Spartina alterniflora*) on channel banks of salt marshes on the east coast of the United States^42^,^43^. At low tide, soils along the creek bank drain more completely than inland soils. Greater subsurface water movement along the creek bank flushes the soil of excess salt and aerates and oxidizes those soils relative to inland soils^43^, promoting vegetation growth. Waterlogged inland soils accumulate sulfides which reduce the growth of vegetation in that area^44^. A high geometric efficiency—corresponding to a low unchanneled path length—means the fresher, oxidized region near channels covers a relatively larger area of the platform. Therefore, an efficient network should support more productive vegetation. Vegetation stabilizes the channel banks, which allows for the development of meanders^10^, while vegetation also concentrates flow into the channels, maintaining their depth by removing sediment that would otherwise accumulate^3^. Meanders snaking across the marsh surface reduce the unchanneled path length, creating an efficient network. We hypothesize that this positive feedback between vegetation and network geometry, mediated by hydrodynamics and biogeochemistry, reinforces the two classes of network geometry seen here. Further investigation of the biogeochemical function of the unvegetated systems and its relation to their network geometry is necessary to resolve the strengths of these feedbacks and their dynamics.

**Figure 2:**
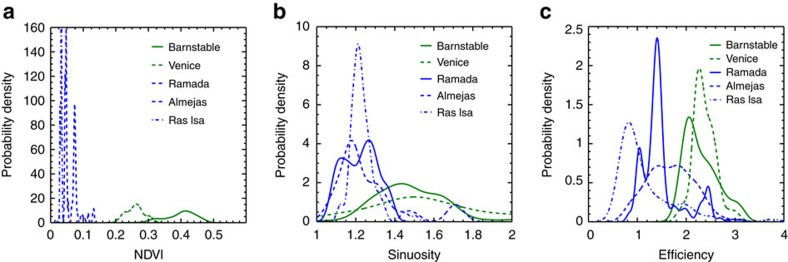
Distributions of ecogeomorphic variables in the five systems. (**a**) Normalized Difference Vegetation Index (NDVI), (**b**) sinuosity, (**c**) geometric efficiency, *l*_h_/*l*. The unvegetated systems (Ramada, Ras Isa and Almejas) are shown in blue and the vegetated systems (Barnstable and Venice) are in green. The distributions of each of the three variables in Ramada and Ras Isa are centred on lower values. These two systems are unvegetated, straight and inefficient. For Barnstable and Venice, the distributions are centred on higher values, so these two are vegetated, sinuous and efficient channel networks. The distributions for the partially vegetated Almejas sit between the vegetated and unvegetated endmembers. The distributions for sinuosity and efficiency in the unvegetated and vegetated systems overlap so that there are individual vegetated subbasins in Barnstable and Venice that are straight and inefficient, while there are unvegetated subbasins in Ramada, Ras Isa and Almejas that are sinuous and efficient.

The relationships between vegetation density (as measured by NDVI), sinuosity of the channels, and efficiency over the five systems (Barnstable, Venice, Ramada, Almejas, Ras Isa) are consistent with the vegetation-network geometry feedback we propose (Fig. 2). Each of the three measures is highest in the two vegetated sites (Barnstable and Venice) and lowest in the unvegetated sites (Ras Isa and Ramada), with Almejas in between. The Barnstable and Venice channel networks are vegetated, sinuous and efficient, while that of Ramada and Ras Isa are unvegetated, straight and inefficient. Almejas has both vegetated and unvegetated surfaces, so the distribution of each of NDVI, sinuosity and efficiency for the Almejas system represent a mixture of a vegetated, Barnstable-like, distribution and an unvegetated, Ramada-like, one.

**Figure 3:**
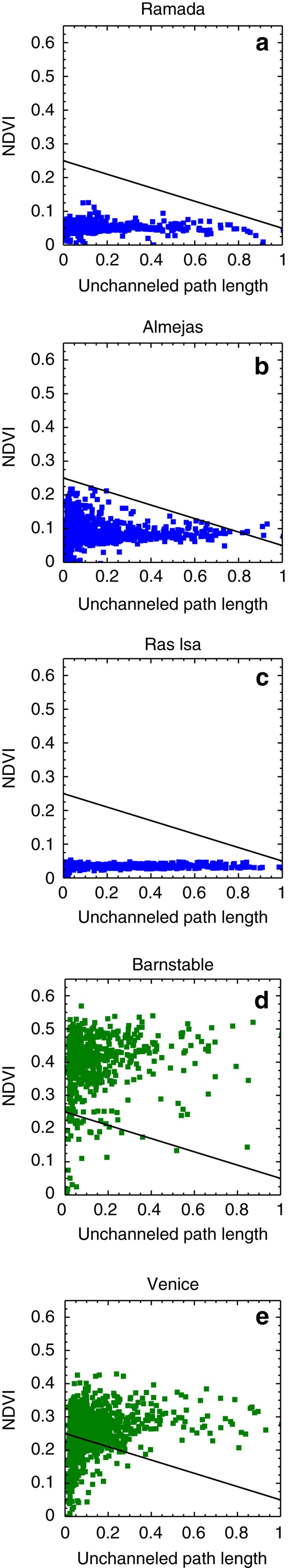
Relationship between unchanneled path length and vegetation density. The values of the NDVI field plotted against the corresponding values of a non-dimensionalized path length field (the path length divided by the maximum path length in the system) for (**a**) Ramada, (**b**) Almejas (**c**) Ras Isa, (**d**)Barnstable and (**e**) Venice. The line (identical on all three plots) divides the plane into two regions. Ramada, Ras Isa and Almejas occupy the lower-left half-plane, meaning no parts of these two platforms have both high vegetation density and high unchanneled path length reflecting the stress on plants in these salt environments. The vegetation that does exist in the Almejas system lies close to channels. In Barnstable and Venice, however, there are areas relatively far from channels with vegetation.

Since sinuosity and efficiency are related across the three marshes, we could feasibly use either one as an indicator of the channel network structure. We prefer the efficiency because it captures the hydrological and biogeochemical processes, which ultimately control spatial variations in plant physiology. If we compare the NDVI and the unchanneled path length (which largely controls efficiency) for Almejas, we find that almost all (99.8%) of the marsh area is either vegetated with low unchanneled path lengths or unvegetated (Fig. 3b). Areas with high unchanneled path lengths never display high NDVI, because the biogeochemical mechanisms identified above make it impossible for vegetation to thrive in those regions. We note that in Ramada and Ras Isa, there are no vegetated regions regardless of path length, while in Barnstable and Venice, there do exist vegetated regions far from channels (Fig. 3a,c). The sinuosity is also a measurement at the scale of a single channel, while the efficiency characterizes the network as a whole. Moreover, a single sinuous channel without bifurcations (for example, a terrestrial river in a floodplain) can display a very low efficiency, because areas far from the channel are not connected. Both meanders and bifurcations are needed to form an efficient network. The key challenge in making the connection between sinuosity and network geometry is to scale local controls on sinuosity and meander and bank evolution^45^,^46^,^47^, including vegetation status, into network statistics like the efficiency.

While there is a definite difference in geometric efficiency between the vegetated Barnstable and Venice systems and the unvegetated Ramada and Ras Isa ones (with the partially vegetated Almejas sitting between the two groups), a wide range of efficiency values is present both within the subbasins of each marsh and within the two groups (Fig. 2c). Like many investigations along these lines^48^,^49^, we find that geomorphological measures are unlikely to be diagnostic of the presence or absence of vegetation, especially at small scales. Simply finding a subbasin with a high efficiency does not imply that the entire marsh is efficient. Processes including hydrodynamics, sediment characteristics and the geological and human histories of the marsh also play a role in shaping channel networks^24^,^50^,^51^, so efficiency is surely not a unique signature of the presence of vegetation on these platforms. Differences in sediment size can influence the cohesive properties of the soil, while differences in the tidal prism and flow conditions may alter the structure of drainage on the platform. The concentrated salinity in arid coastal soils reduces the strength of that soil^37^. However, high soil salinity is also toxic to vegetation so that the impact of vegetation on the soil strength is not readily separated from that of soil salinity with the data such as those presented here.

While sabkhas and hypersaline lagoons are common on arid coasts, sabkhas, which are both unvegetated and channelized, such as the Ras Isa system and the esteros of Ramada and Almejas are relatively rare. They require a unique combination of a supply of appropriate sediment and the right geomorphic setting to build-up an intertidal platform, a large enough tidal prism to develop a channel system and a climate dry enough to concentrate salinity so that vegetation is unable to grow on the platform. Most sabkhas either have not accumulated enough sediment to build a platform (like the Khor Umm Al Qiwen, United Arab Emirates^28^), are supratidal or otherwise not hydraulically connected to the ocean (such as the Sabkha Boujmel, Tunisia^52^) or are vegetated (as in the Khnifiss Lagoon, Morocco). When the conditions exist to form a channelized, unvegetated platform, the lack of vegetation is associated with a network geometry different enough to allow us to differentiate these channel networks from those of vegetated systems including salt marshes. Efficient tidal channel networks may not be a distinct topographic signature of life^48^, but they do reflect the biotic processes that shape the landscape.

## Methods

### Measures of network geometry

Horton^25^ defined the drainage density (*D*) of a basin as the ratio of channelized network length to watershed area. It appears that the drainage density of tidal channel networks is roughly constant^24^, which suggests that Hortonian measures cannot distinguish channel networks with different meandering or branching characteristics^24^,^53^,^54^. More appropriate is a measure of the distance a particle of water at a point on the marsh platform travels before reaching a channel (the unchanneled path length, *l*)^24^,^26^. This measure defines a scalar field over the entire area of the marsh platform. On subdividing the basin into a number of subbasins, the mean of the unchanneled path length on every point on the surface of the platform can be found for each individual subbasin. Dividing the inverse of drainage density (the Hortonian length scale, *l*_h_) by the mean unchanneled path length for each subbasin provides a nondimensional measure of the efficiency with which, for a given subbasin area and network length, the channel system serves the surface of the marsh platform. This efficiency is dependent on the branching and meandering characteristics of the network that the drainage density alone fails to capture.

### Image processing and network topology

Channels were extracted manually from satellite imagery (Google Earth; Barnstable: 11 March 2012; Ramada: 10 December 2003; Almejas: 15 February 2004; Ras Isa: 19 July 2013) of the Ramada, Barnstable, Ras Isa and Almejas systems. The Venice channel map is that used by Fagherazzi *et al*.^54^. We define drainage directions on the marsh platform by generating a time-averaged water surface topography on the platform of the salt marsh^55^. This procedure simplifies the relevant flow equations to the solution of a Poisson problem


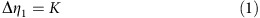


where *η*_1_ is the water surface topography and *K* is a constant factor accounting for friction and the average water depth on the marsh platform, and which is here set equal to 1. The solution of this equation is a boundary value problem on a domain, Γ, corresponding to the marsh platform with boundary ∂Γ corresponding to both the channels and the boundary between the marsh and the upland. The boundary conditions used are that *η*_1_=0 on the channels and the normal derivative, *∂η*_1_/*∂***n**=0 on the upland-marsh boundary. The former uses the assumption that tidal waves propagate much faster within the creek than on the platform, making the water surface effectively horizontal within the creeks. The latter boundary condition imposes a no-flux condition on the upland-marsh boundary. We refer the reader to Rinaldo *et al*.^55^ for a more complete derivation of this equation. The Poisson problem was solved over the digitized channel network (Supplementary Fig. 1) using a successive over-relaxation scheme with a five-point Laplacian stencil.

The gradient of the water surface topography reflects the average direction of flow on the marsh platform. We use the topographic gradient to derive flow lines from each point on the platform to a channel. The unchanneled path length is the length of each of these flow lines (Supplementary Fig. 2). With suitably labelled channels, we can delineate watersheds of each channel (which we call the ‘subbasins') as the collection of points whose flow lines terminate on that channel.

To label the channels properly and develop the network topology such that each channel could be associated with its tributaries, we first calculate an approximate centerline to the channel network using a thinning algorithm^56^. The thinning algorithm produces a four connected skeleton, so each channel pixel has exactly one incoming and one outgoing neighbour unless that pixel is an endpoint of the network (in which case it has only an outgoing neighbour) or a node representing the confluence of two or more channels (in which case it has several incoming neighbours and—usually—one outgoing neighbour). We performed a depth-first search on the skeleton starting from the inlet and using this rule to identify branch points and individual channel segments which are then labelled appropriately for the watershed delineation procedure outlined above.

Within each subbasin, the subbasin area, the network length as measured along the centerline of the channel, and the mean unchanneled path length were calculated. The ratio of the two former measures provides the Hortonian length (*l*_h_), and the ratio of Hortonian length to mean unchanneled path length for an individual basin gives the geometric efficiency of that basin. Having the network topology represented as a graph allows us to calculate the statistics over many scales. For instance, for a channel that collects two first-order streams, we can calculate the area of the subbasin as the area of the subbasins of each of its tributaries plus the area of the platform, which drains directly into the channel segment. Sinuosity is defined as the ratio of channel length along the centerline to straight-line distance from the start to the end of the channel. This means each path within the channel network from the tip of a first-order stream to the inlet has its own sinuosity. We therefore calculate the average sinuosity of channels within each subbasin for higher-order channels. We use the Normalized Difference Vegetation Index to estimate the vegetation characteristics of each of the systems. NDVI is defined as


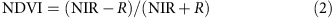


where NIR is the reflectance in the near-infrared portion of the spectrum and *R* is the reflectance in the red portion of the spectrum.

We use Landsat 8 Operational Land Imager imagery, which was upsampled by sampling the Landsat image at the locations of pixels in the channel network imagery so that the resolution of the resampled Landsat imagery matched the resolution of the channel network imagery (Barnstable: 2.6 m per pixel; Ramada: 5.9 m per pixel; Almejas: 3.2 m per pixel; Venice: 1.7 m per pixel; Ras Isa: 0.568 m per pixel). NDVI and the unchanneled path length are the two statistics, which we calculate over the entire platform surface. Figure 3 shows the correlation between these two measurements on the platform and Supplementary Fig. 3 shows the same correlation when the measurements are averaged to the subbasin scale.

### Variability in efficiency

We note the high amount of variance in efficiency in each of the three systems, more pronounced for the Almejas and Ramada networks (Fig. 2, Supplementary Fig. 3). Some of that variance can be explained by the specific watershed delineation algorithm. Small spurs off of the main channel can be interpreted by the thinning algorithm as individual channels when they may not represent a geomorphically distinct feature. These spurs, depending on their position on the platform, can occasionally end up with a relatively large contributing area as they intercept much of the flow that would otherwise head into the main channel. As a result, they often have relatively high Hortonian lengths and high efficiencies that do not necessarily reflect the actual structure of the channel network. One way to reduce some of this variance is therefore to consider only higher-order subbasins. The errant spurs are usually first-order streams in the calculation and by focusing only on the higher-order subbasins, we effectively average out the contribution of the spurs to the network statistics. In Fig. 2, we therefore only show the distributions of efficiency, sinuosity and NDVI for subbasins with Strahler stream order greater than 2. The overall pattern—the increasing efficiency with increasing NDVI and sinuosity—is repeated for all subbasins (Supplementary Fig. 4), but we believe that the statistics for higher-order basins more accurately reflect the actual channel network structure.

All of the above analysis was conducted using the Julia programming language^57^.

### Data availability

The data and software that support the findings of this study are available from the corresponding author on request.

## Additional information

**How to cite this article:** Kearney, W. S. *et al*. Salt marsh vegetation promotes efficient tidal channel networks. *Nat. Commun.* 7:12287 doi: 10.1038/ncomms12287 (2016).

## Supplementary Material

Supplementary Information Supplementary Figures 1-4

## Figures and Tables

**Table 1:** Channel network properties.

**Marsh**	**Tidal range (m)**	**Vegetation**	***D*** **(m**^−1^)	***l***_**h**_ **(m)**	***l*** **(m)**	***l***_**h**_**/*****l***
Barnstable	3	Vegetated	0.011	86.63	45.44	1.91
Venice	0.7	Vegetated	0.016	60.91	20.50	3.15
Almejas	7	Mixed	0.0075	133.02	74.57	1.41
Ramada	7	Unvegetated	0.0069	145.30	132.98	1.09
Ras Isa	1	Unvegetated	0.016	62.24	82.53	0.75
