# Supplementary material for: Salt marsh vegetation promotes efficient tidal channel networks
Source: Nat Commun. 2016 Jul 19;7:12287. doi: 10.1038/ncomms12287 (PMC4960299; doi:10.1038/ncomms12287)
Supplement: Supplementary Information — Supplementary Figures 1-4 [file ncomms12287-s1.pdf]

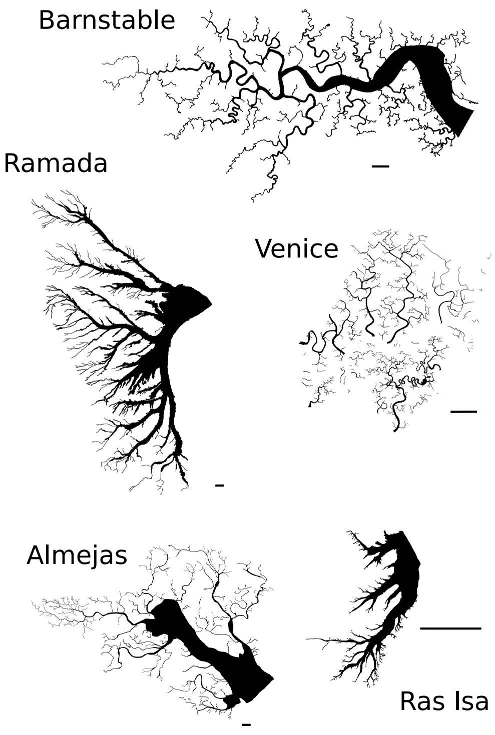

Supplementary Figure 1: **Maps of channel networks.** Channels extracted from satellite imagery (Google Earth, 2013; Fig. 1) of the five intertidal systems studied. The scale bars accompanying each network are 200 m in length.

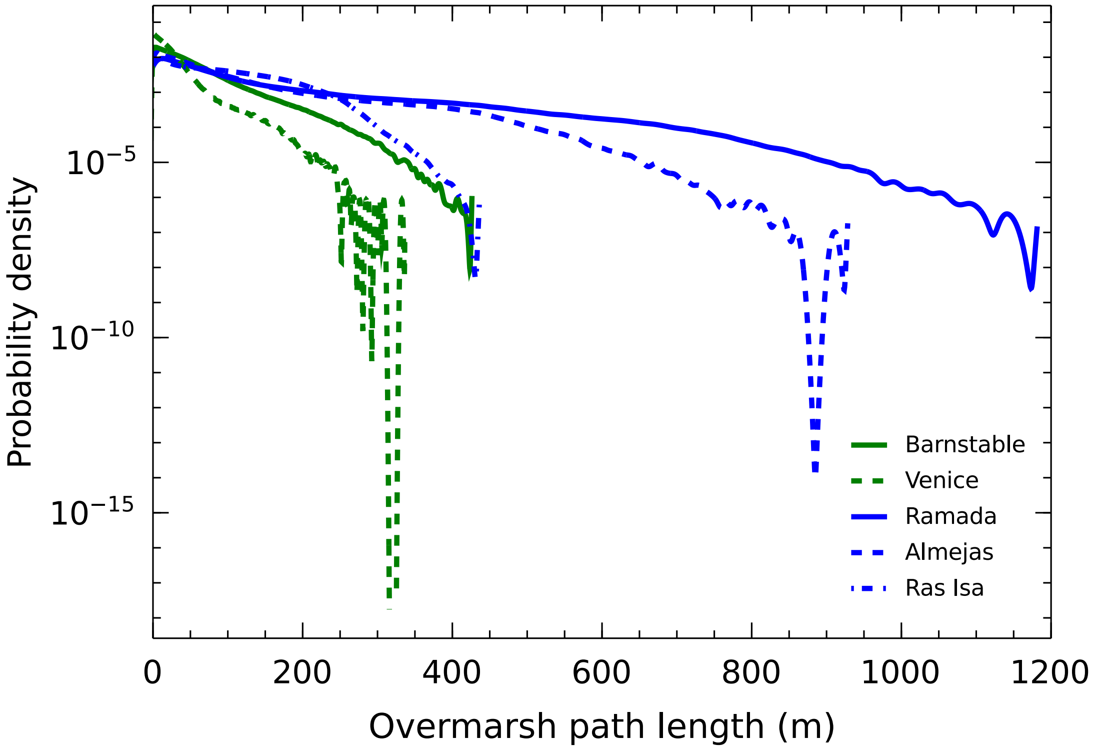

Supplementary Figure 2: **Probability distributions of unchanneled path lengths.** Distributions of unchanneled path lengths in a semilog plot form roughly linear relationships suggesting exponential distributions as in Marani et al. (2003) and Tucker et al. (2001). The slope of the linear relationship is the mean unchanneled path length for the entire system. Venice and Barnstable have the lowest mean unchanneled path lengths (20.50 m and 45.44 m, respectively) while Ramada has the highest (132.98 m) with Almejas in between (94.61 m). Ras Isa has a relatively small unchanneled path length (82.53 m) which is due to its small size.

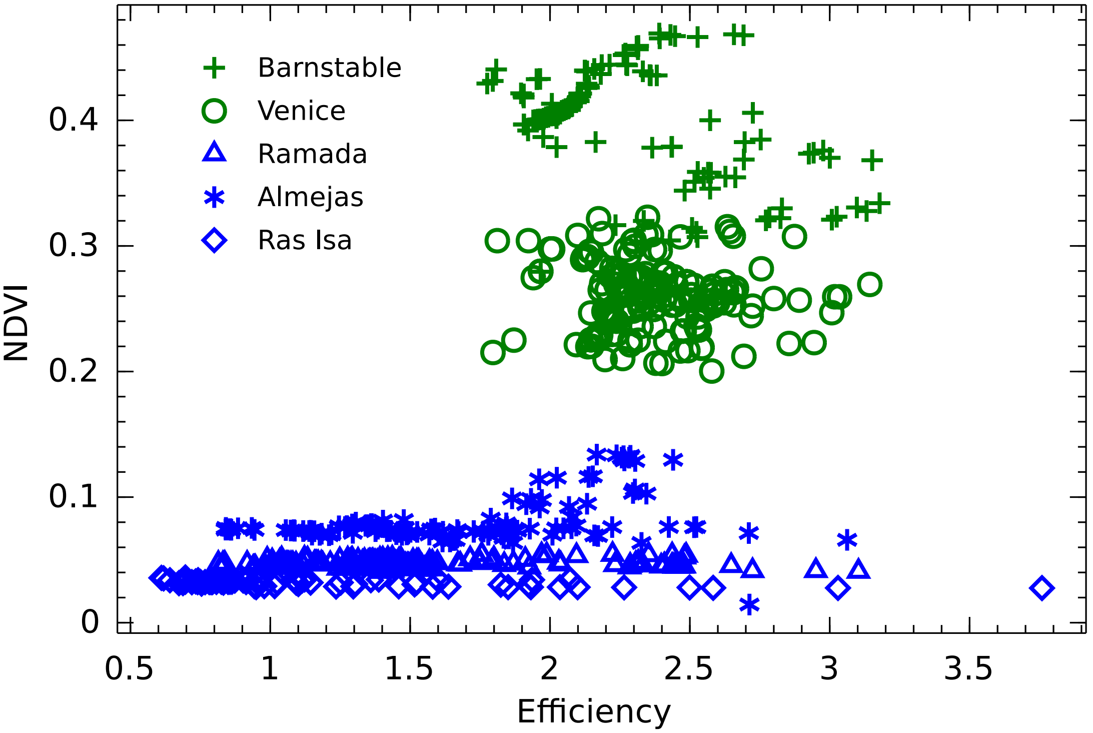

Supplementary Figure 3: **Correlation between NDVI and efficiency.** While network efficiency and vegetation as measured by NDVI are positively correlated across sites (correlation coefficient $r = 0.650$, $p < 0.05$), statistical variability within sites – particularly the high variance of efficiency – demonstrates the need to conduct the efficiency analysis over the whole network rather than individual subbasins.

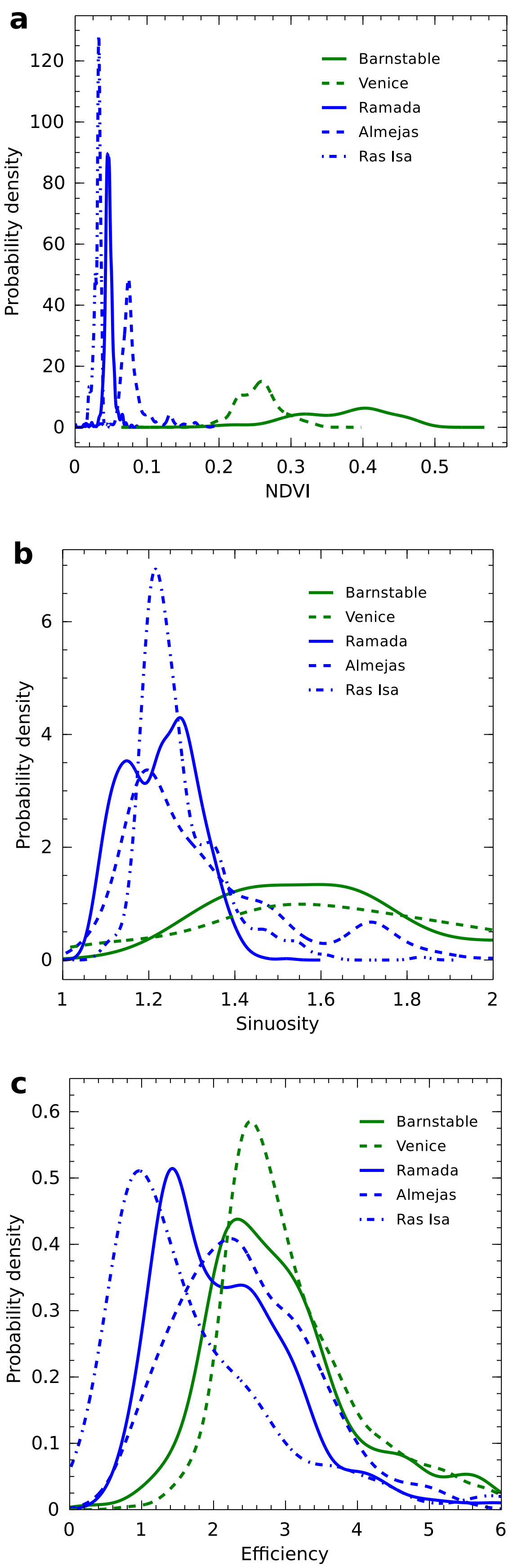

Supplementary Figure 4: **Distributions of ecogeomorphic variables for all subbasins.** Figure is identical to Fig. 2 in the main text except that it shows the distributions of NDVI, sinuosity and efficiency for all subbasins within each system rather than the third- and higher-order basins as in Fig. 2.
